# Cardiac Arrest as the Initial Presentation of Undiagnosed Kawasaki Disease: A Case Report and Literature Review

**DOI:** 10.7759/cureus.40855

**Published:** 2023-06-23

**Authors:** Ashraf Sliem, Alfonso Siu, Jin Zheng, Sergio Magana, Zakaria Alagha, Muhammad Ghallab, Manuel Lopez

**Affiliations:** 1 Internal Medicine, Flushing Hospital Medical Center, Flushing, USA; 2 Internal Medicine, Marshall University Joan C. Edwards School of Medicine, West Virginia, USA; 3 Internal Medicine, Icahn School of Medicine at Mount Sinai, New York City Health and Hospitals, Queens, USA; 4 Cardiology, Flushing Hospital Medical Center, Flushing, USA

**Keywords:** coronary artery aneurysms, vasculitis, undiagnosed kawasaki disease, cardiac arrest, late sequelae of kawasaki disease

## Abstract

Kawasaki Disease (KD) is a self-limited acute vasculitis that mainly affects medium-sized arteries in childhood, with the coronary arteries being one of the main targets. A well-known complication is a coronary aneurysm with myocardial ischemia. We report the case of a 29-year-old female with an insignificant past medical history who presented with sudden cardiac arrest. Labs were significant for elevated troponin, consistent with non-ST elevation myocardial infarction, given diffuse ST depression on the electrocardiogram. The patient underwent a coronary angiogram that revealed diffuse coronary artery disease with multiple ulcerations, aneurysms, and occlusions consistent with KD, despite denying prior history. Cardiac arrest may be the initial presentation of undiagnosed KD and should be considered as one of the differential diagnoses.

## Introduction

Kawasaki disease is an acute, self-limited vasculitis of unknown etiology that primarily affects infants and children younger than five years of age. It is characterized by a high fever, mucocutaneous inflammation, and cervical lymphadenopathy, targeting medium-sized blood vessels. While the prognosis is generally favorable, Kawasaki disease can lead to complications such as coronary aneurysms and myocardial ischemia, which can result in increased mortality during adulthood, with 1%-2% of the patients dying suddenly from acute heart failure [1]. The risk of myocardial infarction appears to decrease one to two years after the acute phase, but adults can still present with complications even after several decades [2,3]. Sudden cardiac arrest as the initial manifestation of Kawasaki disease in adulthood has been rarely reported in the literature.

## Case presentation

A 29-year-old female with no significant past medical history presented after experiencing a witnessed cardiac arrest during her birthday party. She was found pulseless, and chest compressions were immediately initiated. When emergency medical services arrived at the scene, an automated external defibrillator (AED) delivered a shock for an undocumented rhythm, and the return of spontaneous circulation (ROSC) was achieved after seven minutes. Upon arrival at the emergency department, she was normotensive with a blood pressure of 104/67, a heart rate of 97/min, a respiratory rate of 18/min, and a temperature of 35.9°C. On physical examination, she was confused and agitated, not following commands but moving all her extremities spontaneously. The remainder of her exam was unremarkable. The toxicology screen was negative except for an alcohol level in serum of 33 mg/dl and initial lab work, as shown in Table 1.

**Table 1:** Summary of the patient's initial test results ALT: Alanine transaminase; AST: Aspartate transaminase, PO2: partial pressure of oxygen, PCO2: partial pressure of carbon dioxide.

Labs	Value	Reference range
Complete blood count		
Hemoglobin (Hb)	12.6 g/dl	12.0-16.0 g/dL
WBC	13.2 x 10(3)/mcL	4.8-10.8 x 10(3)/mcL
Platelets	256 x 10(3)/mcL	150-450 x 10(3)/mcL
Kidney functions tests		
Blood urea nitrogen	13 mg/dL	6-23 mg/dL
Creatinine	0.9 mg/dL	0.5-1.2 mg/dL
Sodium	139 mmol/L	136-145 mmol/L
Potassium	3.8 mmol/L	3.5-5.1 mmol/L
Liver function tests		
ALT	20 U/L	0-33 U/L
AST	56 U/L	5-32 U/L
Coagulation profile		
D-dimer	10,040 ng/mL	≤285 ng/mL
Activated partial thromboplastin time (aPTT)	31.2 seconds	25.1-36.5 seconds
Prothrombin time (PT)	12.6 seconds	10.0-13.0 seconds
Arterial blood gases (ABG)		
PH	7.34	7.35-7.45
PO2	111 mmHg	83-108 mmHg
PCO2	34 mmHg	32-35 mmHg
Lactate	8.02 mmol/L	0.4-0.8 mmol/L
Troponin-I (1)	0.082 ng/ml	≤0.010 ng.ml
Troponin-I (2)	4.84 ng/ml	≤0.010 ng.ml
Troponin-I (3)	9.68 ng/ml	≤0.010 ng.ml

**Figure 1:**
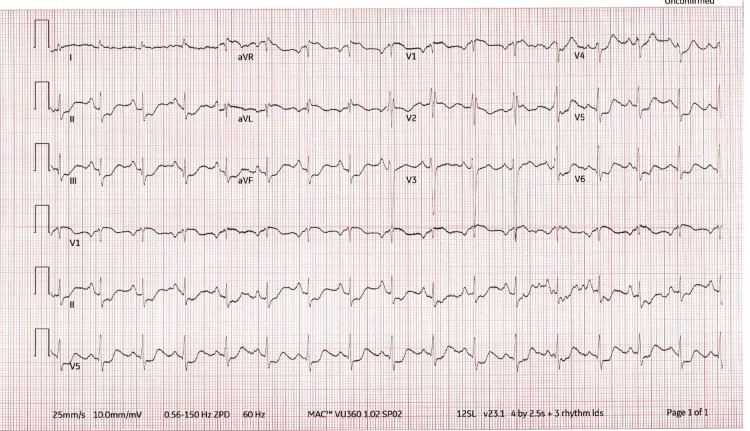
The 12-lead electrocardiogram shows sinus rhythm with diffuse ST segment depressions in leads II, III, aVF, and V4-6 with an incomplete right bundle branch block

An electrocardiogram (EKG) (Figure 1) revealed diffuse ST segment depressions in leads II, III, aVF, and V4-6 with an incomplete right bundle branch block.

CT angiography of the chest was negative for pulmonary embolism but showed bilateral lower lobe consolidation suggestive of atelectasis. While in the emergency department, the patient was intubated for airway protection and admitted to the cardiac intensive care unit. She was administered aspirin (325 mg), clopidogrel (300 mg), atorvastatin (80 mg), and enoxaparin (1mg/kg). A bedside point-of-care echocardiogram showed apical hypokinesia with preserved ejection fraction. These findings were confirmed in a few hours by a transthoracic echocardiogram (TTE), which revealed a left ventricular (LV) ejection fraction (EF) of 45%-50%, apical hypokinesia, normal right ventricular (RV) function, and no valvular abnormalities. The following day, the patient was extubated and remained awake but confused. Magnetic resonance imaging of the head yielded negative results for any acute pathology.

The patient subsequently underwent a coronary angiogram, which revealed severe diffuse coronary artery disease (CAD) with multiple ulcerations, aneurysms, and occlusions. The left main coronary artery was moderately ectatic with the severe diffuse disease throughout the vessel. There was a moderate aneurysm in the left anterior descending artery (LAD) with diffuse disease to the extent that the distal LAD was filled by collaterals from the mid-LAD and the second septal branch. The other lesions were as follows: Ostial LAD to proximal LAD lesion 50% stenosed, mid-LAD 100% stenosed, and distal LAD 95% stenosed.

**Figure 2:**
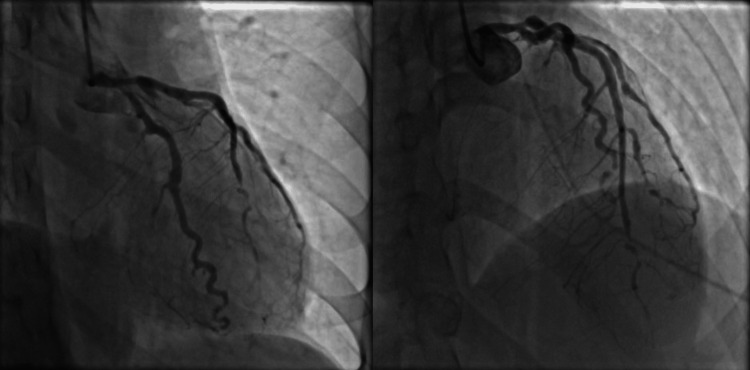
Coronary angiogram showing severe diffuse coronary artery disease with multiple ulcerations, aneurysms, and occlusions.

These findings were consistent with Kawasaki arteritis or other forms of vasculitis (Figure 2).

Further, the workup showed an elevated erythrocyte sedimentation rate of 53 mm/hr, which later normalized, antinuclear antibodies (ANA) at a titer of 1:320, negative double-stranded DNA, anti-Ro, anti-La, c-ANCA, and p-ANCA, cardiolipin, and Beta2 GP1 Ab.

A cardiac MRI was obtained to assess myocardial viability, which was normal, with normal left ventricular (LV) and right ventricular (RV) function. There was no evidence of myocardial scarring.

The patient's mental status recovered back to baseline, and she was offered an implantable cardiac defibrillator for secondary prevention. However, she declined and opted for a wearable defibrillator instead. She was discharged on aspirin 81 mg daily, clopidogrel 75 mg daily, metoprolol succinate 25 mg daily, and atorvastatin 40 mg at night.

She followed up at the outpatient cardiology clinic two weeks later, and a follow-up TTE showed normal LVEF with apical akinesia.

## Discussion

Kawasaki disease is an acute self-limited vasculitis that can lead to coronary artery damage, resulting in progressive atherosclerotic changes and an increased risk of thrombus formation, which can cause structural damage. This damage may remain clinically silent for many years and can sometimes be detected by echocardiogram in approximately 25% of untreated patients [2-4].

In a retrospective study by Burns, 74 cases of presumed sequelae of Kawasaki disease were reviewed [4]. Among them, 40 cases were from English patients, and 34 cases were from Japanese patients. Of the total cases, 57 were male, with a mean age of 24.7 ± 8.4 years. Symptoms on presentation included chest pain/myocardial infarction (60.8%), arrhythmia (10.8%), and sudden death (16.2%). In 82% of these patients, these symptoms were exercise-induced. Angiographic findings revealed coronary artery aneurysms in 93.2% of cases and coronary artery occlusion in 66.1% of cases. Additionally, extensive development of collateral vessels was reported in 44.1% of patients. Autopsy findings showed coronary artery aneurysms in 100% of cases and coronary artery occlusion in 72.2% of cases.

A more recent study conducted in San Diego County demonstrated that 5% of patients younger than 40 who presented with acute coronary syndrome (ACS) had aneurysms secondary to KD during childhood [5]. The study further revealed that the majority of these patients also had at least one additional risk factor for CAD. Among the cases reviewed, it was found that all patients with a known history of KD had significant coronary aneurysms.

Given our patient's initial presentation with sudden cardiac arrest and an unidentified shockable rhythm detected by an AED, the main differential diagnosis included cardiac arrhythmia due to channelopathies or structural heart disease. However, a subsequent coronary angiogram revealed multiple aneurysms along with severe diffuse CAD. These findings are likely attributable to undiagnosed KD vasculitis during her childhood, as no additional risk factors were identified.

## Conclusions

The differential diagnosis of sudden cardiac arrest in young patients poses a challenge. The first-line approach involves a comprehensive assessment for channelopathies, structural heart disease, autoimmune etiologies, and drug abuse. It is important to consider Kawasaki disease sequelae, albeit rare, in young patients. Guidelines recommend annual screening with TTE for known patients with KD to detect potential complications. However, undiagnosed KD may go unnoticed, leading to serious and potentially life-threatening outcomes such as myocardial infarction and cardiac arrest.
